# Most Common Injuries in Resistance Training: Mechanisms, Therapeutic Interventions, and Preventive Strategies

**DOI:** 10.7759/cureus.94035

**Published:** 2025-10-07

**Authors:** Oliwia Kawa, Wojciech Zywiec, Bartosz Czyzewski, Karol Kozlowski, Alicja Dorota, Michal Dorota, Cezary Milczarek, Illia Koval, Anna Mariankowska, Joanna Czyzewska

**Affiliations:** 1 Medicine, Provincial Hospital of St. Luke in Tarnów, Tarnów, POL; 2 Medicine, Central Clinical Hospital of the Medical University of Łódź, Łódź, POL; 3 Anesthesia and Critical Care, Provincial Integrated Hospital in Kielce, Kielce, POL; 4 Medicine, Zaglebiowskie Oncology Center in Dąbrowa Górnicza, Dąbrowa Górnicza, POL; 5 Conservative Dentistry, Central Clinical Hospital of the Medical University of Łódź, Łódź, POL; 6 Medicine, Provincial Hospital in Poznań, Poznań, POL; 7 Rheumatology, Central Clinical Hospital of the Medical University of Łódź, Łódź, POL

**Keywords:** injury prevention, musculoskeletal injuries, rehabilitation, resistance exercise, sports injuries, weight training

## Abstract

Resistance training is a structured exercise modality aimed at enhancing strength, hypertrophy, and overall fitness. However, it carries an inherent risk of musculoskeletal injuries, particularly in the shoulder, lower back, knee, and wrist/hand. The most common injury mechanisms include muscle strains, tendon tears, tendinitis, and sprains, with acute injuries predominating. Key contributing factors are improper technique, excessive loading, insufficient recovery, and anabolic steroid use. While most injuries are mild and resolve with conservative management, severe cases may require surgical intervention. Prevention is paramount, emphasizing proper technique under professional supervision, progressive overload management, and athlete education. Resistance training itself serves as a protective measure against future injuries. Future research should further investigate the role of anabolic steroids in injury risk and refine sport-specific preventive strategies to optimize athlete safety and performance. A literature search of Google Scholar, PubMed, and ScienceDirect databases was performed. Articles were searched in English using the following keywords: "weight training", "resistance exercise injuries", "weightlifting", "weightlifting injuries", and "resistance training injuries". This paper summarizes the data available in the literature on injuries in resistance training. It focuses on illustrating the most common injuries in strength sports disciplines, their mechanisms, treatment methods, rehabilitation, and prevention.

## Introduction and background

**Figure 1:**
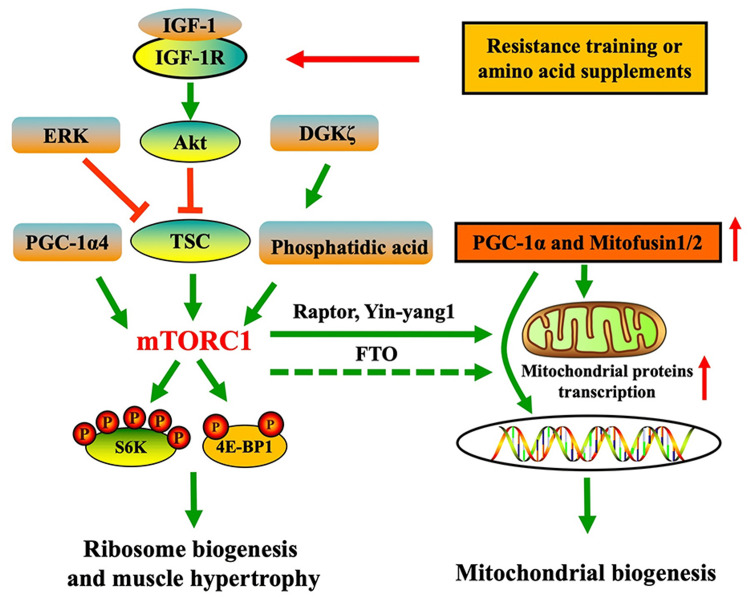
Activation of mTOR and PGC-1α pathways in skeletal muscle following resistance training and nutritional support mTOR: mammalian target of rapamycin; PGC-1α: peroxisome proliferator-activated receptor γ coactivator 1α; TSC: tuberous sclerosis complex; 4E-BP: eukaryotic initiation factor 4E binding protein; S6K1: ribosomal S6 protein kinase 1; ERK: extracellular signal-regulated kinase; Akt: protein kinase B; FTO: fat mass and obesity-associated gene Source: Zhao Y-C, Dual roles of mTOR in skeletal muscle adaptation: coordinating hypertrophic and mitochondrial biogenesis pathways for exercise-induced chronic disease management. Front. Med. 12:1635219. doi: 10.3389/fmed.2025.1635219. This figure is from an open-access article distributed under the terms of the Creative Commons Attribution License (CC BY) [1].

Resistance training is a structured exercise modality that utilizes external loads (e.g., free weights, machines, or body weight) to induce mechanical tension and metabolic stress on skeletal muscles. It typically involves structured repetitions and sets targeting specific muscle groups. This stimulus triggers myofibrillar hypertrophy, neuromuscular adaptations, increased protein synthesis via the mTOR pathway activation, and an increase in connective tissue (Figure 1) [1]. The systematic increase in resistance, volume, or intensity (progressive overload) is a fundamental principle driving strength gains (force production capacity) and muscular hypertrophy [2].

Resistance training can be performed by amateurs to improve physical fitness, enhance physique, or for rehabilitation after injuries and surgeries. Some athletes practice resistance training professionally by participating in sports competitions across various disciplines. These competitions, such as weightlifting, powerlifting, bodybuilding, strongman, Highland Games, and CrossFit, differ from one another, sometimes significantly, but all are based on strength exercises involving significant weights.

Olympic weightlifting focuses on lifting a barbell overhead with two technical lifts: the snatch (one motion) and the clean and jerk (two motions). Powerlifting is a strength sport that tests maximal strength in three lifts: the squat, bench press, and deadlift. Competitors have three attempts per lift to achieve their highest total. Bodybuilding, on the other hand, is an aesthetic-focused discipline aimed at maximizing muscle size, symmetry, and definition. It is judged on physical presentation rather than strength or performance. However, training for bodybuilding competitions largely revolves around weight training and similar techniques as those used in other strength sports. Strongman competitions feature unconventional feats of strength, such as lifting odd objects (e.g., logs, atlas stones), carrying heavy loads, and pulling vehicles. The Highland Games is a traditional Scottish competition that combines strength and skill events, such as the caber toss, stone put, and weight over bar. It blends athleticism with cultural heritage. CrossFit, the newest sport in this category, is a high-intensity fitness program that blends weightlifting, gymnastics, and cardio. Workouts (workout of the day (WOD)) vary daily and test broad fitness across multiple domains, including strength, endurance, and agility.

The aim of this narrative review is to summarize the available literature on injuries in resistance training. It focuses on the most common injuries in strength sports, their mechanisms, treatment methods, rehabilitation, and prevention. This publication aims to increase athletes’ awareness of their health status and the risks associated with training, as well as to draw the attention of physicians, particularly sports medicine specialists and orthopedists, to areas that require special focus in clinical practice when treating patients who participate in these sports. Furthermore, this review critically examines the methodological quality of previous studies, highlighting gaps in sample size, reporting consistency, and prospective monitoring, which are essential for developing evidence-based preventive strategies.

Additionally, this narrative review integrates biomechanical and physiological perspectives to better understand why certain injuries occur during specific exercises, emphasizing the role of joint alignment, muscle-tendon load distribution, and neuromuscular control in injury susceptibility. This mechanistic understanding lays the foundation for developing targeted preventive interventions.

## Review

Frequency and mechanisms of injuries

A contusion (Latin: contusio) is a general medical term referring to damage to the body’s tissues caused by a traumatic mechanical force. From a pathophysiological standpoint, a contusion encompasses injuries to soft tissues, such as muscles, tendons, ligaments, blood vessels, and peripheral nerves, resulting from direct external forces (e.g., impact, fall, compression) that produce morphological and functional changes within these anatomical structures.

According to studies [2-14], the most common sites of injury during resistance training are the shoulder (10.5%-50%), lower back (12.9%-48%), knee (11%-21%), and hand/wrist (17.8%). The underlying pathophysiology in these regions most frequently involves muscle and tendon strains and tears, tendinitis, and ligament sprains, which together account for 46%-60% of cases. Furthermore, the majority of these injuries are acute in nature (59.6%-75%).

In the case of the shoulder, the main cause of injury is the fact that the shoulder complex is not built to bear heavy weights; in fact, it’s considered a non-weight-bearing joint. Another important factor is the incorrect training routine that leads to muscle imbalance. This promotes shoulder instability, which makes this location more prone to injuries. Moreover, the orientation of the shoulder structures that are considered correct in certain exercises might induce further injuries [9,15]. One of the most common injuries is biceps tendon rupture. This can occur during resistance training, especially during exercises involving the lifting of heavy loads. Most traumatic ruptures of the biceps tendon are linked to lifting weights of 68 kilograms or more. The long head of the biceps tendon is the most commonly affected site. Other frequent locations of rupture include the glenoid labrum and the musculotendinous junction [8,16].

Another common injury is rupture of the pectoralis major muscle. This pathology mostly happens during the bench press exercise, mainly because the position requires both upper limbs in abduction and internal rotation, placing the pectoralis major muscle in a lengthened yet actively contracting state as it generates force to lift the weight. During the lowering phase of the movement, the pectoralis major functions eccentrically to control the descent of the weight and prevent it from collapsing onto the chest. However, any mechanical error, muscular fatigue, or imbalance can compromise this control, potentially causing a drop in the weight asymmetrically. Such a sudden, forceful contraction under high tension may result in a rupture injury [17]. Shoulder instability, particularly anterior instability, is another injury that is frequent in resistance training individuals. The primary stabilizing structure of the shoulder is the glenohumeral joint, which maintains the humeral head securely within the glenoid fossa. The stability of the glenohumeral joint relies on a complex interplay between static and dynamic stabilizers. Static stabilizers include the glenoid labrum and the glenohumeral ligaments. The glenoid labrum, a fibrocartilaginous structure, deepens the glenoid cavity and enhances joint congruity, while the superior, middle, and inferior glenohumeral ligaments provide passive restraint against excessive translation of the humeral head. Dynamic stabilizers primarily consist of the rotator cuff muscles, supraspinatus, infraspinatus, teres minor, and subscapularis, which actively control humeral head positioning during movement. Additional muscles, such as the deltoid and scapular stabilizers, contribute to coordinated shoulder function and joint protection. Together, these structures allow for a wide range of motion while minimizing the risk of dislocation and soft tissue injury. Any intrinsic or extrinsic force that displaces the humeral head from its articulation with the glenoid can result in instability. Resistance training often includes exercises that position the upper limbs in abduction and external rotation, positions that promote repeated anterior translation of the humeral head relative to the glenoid. Over time, this repetitive motion can lead to anterior capsular hyperlaxity and, ultimately, anterior shoulder instability [15].

The back, especially the lumbar region, is another very common site of injury. These injuries most often arise from a strength imbalance between the abdominal or lower-limb muscles and the muscles of the lower back. Other contributing factors include improper exercise technique, lifting excessively heavy loads, and overly frequent or intense training sessions, all of which increase the risk of injury in this area. The most common pathophysiological cause of back injury is the strain of a single muscle, accounting for up to 75% of cases [18]. More serious injuries can also occur, typically involving the intervertebral discs, the vertebral bodies’ intersegmental regions, or the vertebral end plates [19]. Resistance training places unique mechanical demands on the lumbar spine, particularly during repeated flexion and extension movements. In flexion, the upper vertebral body tilts upward and translates anteriorly, tightening the posterior fibers of the annulus fibrosus and the supporting posterior ligaments. Conversely, extension reverses these motions, loosening the posterior structures while placing tension on the anterior elements. Because the posterior annulus is both taut and at its structurally weakest point during flexion, most lumbar injuries occur during forward-bending actions [19].

Knee injuries are also common. Resistance training places significant repetitive stress on the knee as it repeatedly flexes and extends under load, most notably during weighted squats. This makes the knee vulnerable to both acute and overuse injuries, the most common of which include iliotibial band syndrome (ITBS), patellofemoral (anterior knee) pain, and meniscal tears [20,21]. 

ITBS is an overuse injury driven by the friction of the iliotibial band sliding over the lateral femoral epicondyle. In resistance training, repeated squats and knee bends under heavy loads cause microtrauma to the iliotibial band, resulting in lateral knee pain, aching, and burning during activity [20].

The patellofemoral joint endures some of the highest compressive forces in the body. Intrinsic risk factors for anterior knee pain include patellar malalignment, genu valgum, and hyperextension, while quadriceps weakness and potential imbalances between vastus medialis and lateralis may also contribute, though definitive evidence is lacking. Extrinsically, the repeated flexion-extension cycles and load-bearing inherent to resistance training further elevate the risk of patellofemoral syndrome [21]. Meniscal tears account for roughly 9.6% of gym-related knee injuries. The menisci distribute load, absorb shock, and aid joint stability and proprioception. In resistance training, excessive axial loads or sudden uncontrolled movements can exceed the meniscus’s mechanical limits, leading to tears [22].

While numerous studies provide prevalence data, many rely on self-reported injuries or retrospective analyses, which may underestimate minor injuries and overestimate severe injuries. There is also variability in reporting definitions of “injury” across disciplines, making cross-study comparisons difficult. Furthermore, few studies integrate biomechanical analyses with epidemiology, limiting understanding of specific causative mechanisms. Prospective studies combining kinematic assessment, load monitoring, and injury surveillance are needed to optimize prevention [13,15].

Treatment, rehabilitation, and the impact of injuries on subsequent functioning

Literature data indicate that a substantial percentage of resistance‐training injuries are relatively mild and do not require medical intervention [5]. Although incidence rates vary slightly between disciplines, the injury profiles are very similar due to the use of comparable or even identical exercises. The vast majority of injuries are strains, overuse syndromes, muscle or tendon tears, and sprains of the musculoskeletal structures.

The initial step in managing tendon tears and ruptures in athletes involves medical evaluation and either modifying the training program to reduce mechanical stress on the affected tissue or immediate medical treatment. Reducing training volume, avoiding aggravating exercises, and substituting with low-impact movements are fundamental strategies. Conservative management often incorporates cryotherapy and analgesics for acute symptom relief. Cryotherapy may reduce pain and swelling during the acute phase of soft tissue injuries [23], although evidence regarding its impact on long-term functional recovery remains mixed. More recently, Racinais and Coudrat [24] confirmed that cryotherapy remains widely applied in sports medicine, particularly for early symptom management, while stressing the need for individualized protocols based on injury type and healing stage.

Electrotherapy methods, such as transcutaneous electrical nerve stimulation (TENS) and interferential current therapy (IFC), play a central role in the conservative management of tendon injuries. TENS remains one of the most evidence-based noninvasive strategies for pain modulation, particularly in chronic conditions such as tendinopathy [25]. IFC is beneficial in reducing both acute and chronic pain associated with soft tissue injuries, suggesting that it may accelerate return-to-play timelines when combined with active rehabilitation [26].

In contrast to cryotherapy, heat therapy is most effective in the subacute and chronic phases of rehabilitation, where it enhances circulation and reduces muscle stiffness. Yutan and Zhang compared heat and cold therapy, showing that heat therapy provides superior relief for delayed onset muscle soreness, a condition with parallels to subacute tendon healing [27]. Localized heat application improves recovery after exercise-induced muscle damage by enhancing muscle flexibility and reducing pain, making it a useful adjunct to progressive loading programs [28].

Emerging evidence supports the use of high-intensity laser therapy (HILT) in tendon and soft tissue injuries. HILT significantly reduces pain and accelerates functional recovery in musculoskeletal injuries [29]. Even low-level laser therapy may improve tissue healing, though higher-intensity modalities appear to produce greater clinical benefits [30]. These findings suggest that laser therapy may serve as a valuable adjunct to conventional rehabilitation in athletes.

Superinductive electromagnetic stimulation (SIS) has recently gained attention as a noninvasive intervention for accelerating muscle and tendon recovery. High-intensity electromagnetic stimulation improves muscle regeneration and functional recovery in athletes [31]. Potential of pulsed electromagnetic field applications in sports medicine, highlighting SIS as a promising innovation that warrants further clinical validation [32].

Pulsed electromagnetic field therapy (PEMF) has been studied extensively for its regenerative and analgesic effects. Noninvasive PEMF therapy effectively reduces pain in musculoskeletal disorders, making it a suitable option for managing chronic tendon injuries [33]. PEMF can enhance tissue healing and functional outcomes in sports-related injuries, particularly when integrated with exercise-based rehabilitation [34].

Some injuries, however, necessitate specialist care, including surgical intervention. Complete tendon ruptures represent a clinical scenario in which conservative treatment is insufficient to restore the structural integrity and biomechanical function of the musculotendinous unit. In such cases, surgical intervention remains the gold standard. The most frequently employed procedures involve direct reattachment of the tendon to its anatomical footprint or end-to-end suturing of the ruptured fibers, often supported by the use of anchors or grafts when native tissue quality is compromised [16,17]. Clinical studies on ruptures of the pectoralis major and distal biceps tendon consistently report that surgical repair enables a significantly higher rate of return to pre-injury activity compared with non-operative management [35,36]. Calhoon and Fry reported in their weightlifting studies that 90.5% of all injuries forced athletes to rest for less than one day, and only 0.5% lasted more than three weeks [3]. In contrast, Raske and Norlin found that 93% of shoulder injuries, 85% of lower-back injuries, and 80% of knee injuries produced symptoms lasting more than four weeks [37]. Kulund and colleagues observed that 33% of injuries caused no impairment, 30% lasted one day to two weeks, 34% lasted between two months and two years, and 5% persisted for more than two years [38]. Taken together, these findings suggest that most resistance‐training injuries do not have lasting negative effects or require extensive medical care, often resolving after just a few days of rest [7].

Early operative repair is generally recommended, as delayed surgery has been associated with tendon retraction, muscle fatty infiltration, and impaired postoperative outcomes [39]. Moreover, surgical reconstruction not only restores mechanical strength but also improves proprioceptive function, thereby facilitating a more complete recovery. Nonetheless, successful outcomes require integrated postoperative rehabilitation protocols, as progressive loading and neuromuscular retraining are critical for minimizing the risk of re-rupture and ensuring long-term durability of the repair.

Critically analyzing these studies, it becomes evident that most injury surveillance is retrospective, and recovery timelines are often self-reported. The lack of long-term follow-up and standardized assessment of functional outcomes limits our understanding of the true impact of injuries on future performance and joint health. Moreover, there is limited stratification by athlete experience, load intensity, and adherence to rehabilitation protocols, which could significantly influence recovery trajectories. Prospective cohort studies with objective functional assessments are needed to validate these findings and optimize rehabilitation strategies [8,13,15].

Additionally, integrating biomechanical evaluations, such as motion capture or force plate analysis, can help identify high-risk movement patterns that predispose athletes to injury. Such data can inform individualized rehabilitation programs that correct deficits in joint alignment, muscular activation timing, and proprioception, ultimately reducing recurrence risk [2].

Injury prevention

Preventing injuries is the most important factor in maintaining athletes’ functional capacity. Because injuries occur relatively frequently, it is essential to follow guidelines that reduce not only their incidence but also their severity.

The first element to focus on is correct exercise technique and appropriate load selection. As Shim et al. point out, injuries happen markedly less when there is supervised coaching [14], suggesting that having a professional trainer oversee technique and programming is a key injury-prevention measure. Source indicates that roughly 46% of resistance‐training injuries result from dropping weights at the end of a set [19], and another 30% arise from excessive microdamage caused by training the same muscle groups too frequently. We can therefore conclude that it is highly recommended, before beginning resistance training, to seek advice from a specialist. If every trainee received clear, expert guidance on how to train safely, both during and between sessions, we could potentially reduce resistance‐training injuries by more than 70%. Thus, every physician, physical therapist, or other specialist working with resistance‐training athletes should prioritize patient education.

Equally important is proper management of injuries, which is crucial for the recovery process. 

In our view, another important, but still insufficiently studied, factor is the use of anabolic steroids and their impact on injury risk. Several studies have suggested that anabolic‐androgenic steroid use is a risk factor for injuries among weight‐training athletes. For example, Sollender et al. described four cases of distal triceps brachii ruptures in patients who used anabolic steroids, and Castro Pochini et al. reported that 96% of participants who suffered a bench‐press-related injury had also used anabolic‐androgenic steroids [39]. This indicates a need for prospective studies evaluating the dose-response relationship between steroid exposure and tendon/muscle injury. Understanding the underlying biological mechanisms, such as steroid-induced alterations in tendon collagen synthesis, muscle stiffness, and impaired tendon healing, would allow clinicians to provide more targeted counseling and preventive measures [1,13].

Given these findings, there remains a pressing need for further research on this topic. Resistance training itself also plays a significant role in injury prevention, especially among young athletes. Properly designed and supervised resistance‐training programs are safe and effective for children and adolescents. Regular participation in these programs reduces injury risk. Key elements of a successful resistance‐training regimen include the presence of experienced coaches or instructors, correct exercise execution, and the adjustment of loads to the individual’s age and level of experience [40].

Furthermore, incorporating dynamic warm-ups [41,42], neuromuscular control exercises [43,44], balance training [45,46], and progressive load periodization [47,48] has been shown to reduce both acute and overuse injuries. Research suggests that athletes following structured prevention protocols demonstrate improved joint stability, lower incidence of tendonitis, and enhanced muscular endurance, all contributing to long-term performance sustainability.

Finally, sport-specific injury prevention programs, tailored to the unique demands of weightlifting, powerlifting, CrossFit, and strongman, remain a critical research gap. Developing evidence-based guidelines for exercise selection, volume management, and biomechanical assessment could substantially reduce injury rates and enhance athlete longevity.

## Conclusions

Resistance training is a highly effective method for improving strength, hypertrophy, and overall fitness, but it carries an inherent risk of contusions and musculoskeletal injuries. The most common injury sites include the shoulder, lower back, knee, elbow, and wrist/hand, primarily due to improper technique, excessive loading, and insufficient recovery, and, as some papers suggest, anabolic steroid usage. Most injuries are mild and resolve with conservative management (rest, physical therapy, and load modification). 

Prevention remains the cornerstone of injury mitigation in weight training. Key strategies include proper technique under professional supervision, gradual progression in loading under expert guidance, and education on safe training practices. Notably, while disciplines like CrossFit may report higher injury rates due to their dynamic nature, structured weightlifting and powerlifting exhibit lower incidences when performed correctly. Additionally, resistance training itself has a protective effect against future injuries by strengthening muscle power and improving joint stability.

In summary, weight training-related injuries are typically manageable, but their prevention hinges on disciplined training practices. Future research should focus on long-term, prospective injury monitoring, steroid-related risk mechanisms, and the development of sport-specific preventive protocols, including neuromuscular conditioning, dynamic warm-ups, and load management, to enhance athlete longevity and performance. Due to the limited number of studies on injuries and small experimental groups, it is necessary to conduct extensive, well‐designed research that would undoubtedly provide a wealth of valuable information.
